# Application of autologous platelet rich plasma in Sun's procedure for acute type A aortic dissection under moderate hypothermia

**DOI:** 10.3389/fcvm.2025.1508188

**Published:** 2025-02-19

**Authors:** Kaiyue Sun, Ruyuan Wei, Zihua Liu, Xin Zhao, Kai Liu

**Affiliations:** ^1^ Department of Cardiovascular Surgery, Qilu Hospital, Shandong University, Jinan, Shandong, China; ^2^ Cheeloo College of Medicine, Shandong University, Jinan, Shandong, China

**Keywords:** acute type A aortic dissection, Sun's procedure, autologous platelet rich plasma, platelet activation, coagulation, inflammation

## Abstract

**Background:**

Coagulopathy and inflammatory response are the intractable complication during Sun's procedure for type A aortic dissection (AAD). This study aims to investigate the efficacy of autologous platelet rich plasma (aPRP) on the patients undergoing Sun's procedure under moderate hypothermia.

**Methods:**

A total of 372 AAD patients who underwent Sun's procedure under moderate hypothermia were divided into aPRP group (aPRP was separated before heparinization and transfused after protamine neutralization) and Non-aPRP group (without aPRP apheresis). Preoperative characteristics, intraoperative data, postoperative outcomes, and perioperative laboratory reports were collected and analyzed.

**Results:**

The operation time (301.1 ± 21.3 vs. 318.1 ± 29.9, *P* < 0.001), postoperative ventilation time [25.3[19.2, 37.0] vs. 31.9[25.4, 43.1], *P* < 0.001] and cardiac intensive care unit stay [4.8[3.5, 7.9] vs. 8.7[4.9,11.2], *P* < 0.001] in aPRP group were significantly shorter than that in Non-aPRP group. Intraoperative blood loss (637.2 ± 24.9 vs. 908.4 ± 51.0, *P* < 0.001), transfusion of allogeneic blood products (PLT: 2.11 ± 1.03 vs. 2.52 ± 0.83, *P* < 0.001; Plasma: 405.6 ± 55.6 vs. 421.0 ± 61.7, *P* *=* 0.012; Cryoprecipitate: 9.7 ± 2.4 vs. 10.4 ± 1.9, *P* *=* 0.002; RBC: 422.7 ± 64.9 vs. 479.2 ± 81.0, *P* < 0.001) and the incidence of postoperative pulmonary complications (8.2% vs. 16.2%, *P* = 0.027) were reduced in aPRP group. The costs of both blood products (9,202.2 ± 1,597.4 vs. 10,031.9 ± 3,471.8, *P* = 0.003) and the total hospitalization (243.5 ± 33.1 vs. 297.6 ± 43.5, *P* < 0.001) were decreased in aPRP group. Furthermore, intraoperative and postoperative levels of C-reactive protein and Interleukin-6 (*P* < 0.001) in aPRP group were lower than that in Non-aPRP group. There was no significant difference in renal, cerebral complications and hospital stay between the two groups.

**Conclusion:**

Application of aPRP in Sun's procedure reduced the perioperative blood loss and allogeneic blood transfusion, contributed to the decreased postoperative pulmonary complications and shortened intensive care unit duration. Apheresis and re-infusion of aPRP in Sun's procedure alleviated postoperative inflammation to a certain degree and was a desirable approach for AAD patients.

## Introduction

Acute type A aortic dissection (AAD) is a fatal disease and open surgery is the optimal therapy for patients. Currently, the main treatment standard of AAD in China is Sun's procedure, which refers to aortic root repairing, arch replacement by four-branched graft and implantation of a trunk stent-graft (1–3). Traditionally, completing the surgery requires prolonged cardiopulmonary bypass (CPB) and deep hypothermia circulatory arrest (DHCA), which lead to dramatic destruction of coagulation system (4, 5). Actually, from the onset of AAD, platelet (PLT) is already activated and massively consumed owing to the influx of blood into the false lumen (6, 7). As we all know, the implanted artificial graft and stent-graft will keep consuming PLT and coagulation factors after Sun's procedure (8, 9). Consequently, AAD patients underwent Sun's procedure are frequently accompanied by intractable hemorrhage and subsequently massive transfusion of allogeneic blood during operation, which is not only places a severe strain on local blood bank (10–12), but also closely related to a variety of adverse reactions. It has been reported that cardiac surgery accounted for 10% of all blood product usage (13). A small percentage of high-risk patients, especially the AAD patients, could account for up to 80% of all blood product usage in the patients undergoing cardiac surgery (10, 11). Previous study have demonstrated the positive and significant correlations between inflammatory cytokines and activation of PLT in AAD (14). The over-activation of PLT in AAD not only impairs the coagulation but also aggravates the acute inflammatory cascade response secondary to aortic dissection. Therefore, it is particularly important to reduce PLT activation and avoid PLT damage in Sun's procedure.

Many strategies have been adopted to decrease the allogeneic blood transfusion in cardiac surgery, including autologous blood donation preoperatively, hematogenesis agents, intraoperative hemodilution, minimized CPB circuit and autologous PLT rich plasma (aPRP) apheresis (10, 15). However, some blood saving strategies such as hematogenesis agents and preoperative blood donation are not suitable for AAD patients. The application of aPRP in cardiac surgery was first described by Harke in 1977 (16) and some other studies were followed (17–19). Autologous PLT rich plasma apheresis aims to minimize the perioperative bleeding in many types of surgeries, and subsequently reduces the allogeneic blood transfusion. However, the benefits of aPRP in these studies still remained controversial, and the efficacy of aPRP in Sun's procedure under moderate hypothermia was reported seldomly in the literature. We hypothesized that aPRP procedure removes the PLT preoperatively and protects them from damage or activation during CPB; and aPRP infusion near the end of the operation could provide a large of intact PLT for coagulation and the protection of PLT might alleviate the inflammation in AAD.

Du et al. compared the incidence of severe systemic inflammatory response syndrome (sSIRS) in AAD patients who underwent total aortic arch replacement under either moderate hypothermia circulatory arrest (MHCA) or DHCA conditions (20). The results showed no statistically significant difference between the two groups. However, in recent years, the continuous development of CPB concepts and cerebral protection strategies, coupled with a deeper understanding of the negative impacts associated with DHCA (21–23), have led to a notable increase in the application of MHCA in aortic surgery. This shift is attributed to the benefits of MHCA, which include shorter CPB times, reduced hospital stays, and lower rates of pulmonary infection and gastrointestinal bleeding compared to DHCA. In our study, all patients underwent surgery with MHCA. We apply a retrospective cohort study to reveal the effects of aPRP apheresis on the AAD patients undergoing Sun's procedure with MHCA.

## Methods

### Patients population

**Figure 1:**
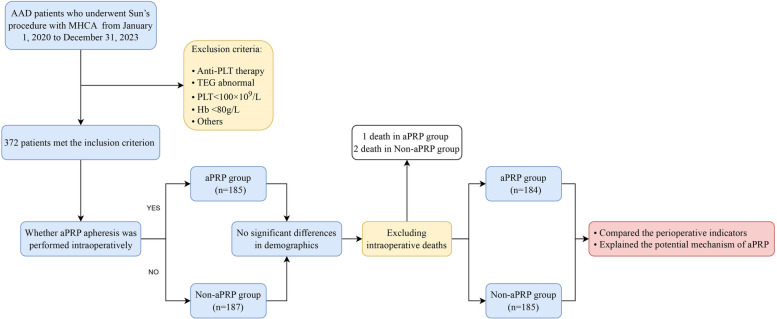
Sketch design of the patient's distribution. AAD, Type A aortic dissection; MHCA, moderate hypothermia circulatory arrest; aPRP, autologous platelet rich plasma; PLT, Platelet; TEG, thromboelastography; Hb, hemoglobin.

The study involving humans was approved by the Institutional Review Board of Qilu Hospital, which was also approved the data analysis for this retrospective study and waived the need for patient consent (Approval number: XWK20190714-01). From January 1, 2020 to December 31, 2023, 372 AAD patients undergoing Sun's procedure with MHCA. Patients were divided into two groups based on whether aPRP apheresis was performed intraoperatively: the aPRP group (*n* = 185, where aPRP was separated before heparinization and transfused after protamine neutralization) and the Non-aPRP group (*n* = 187, without aPRP apheresis). The inclusion criterion mainly involved two aspects: (1) AAD was diagnosed by the spiral computed tomography angiography examination; (2) the patients were scheduled for emergency surgery, which was defined as the operation performed within 8 h after admission. In view of the importance of the PLT function in Sun's procedure, the exclusion criteria were as follows: (1) anti-PLT therapy before surgery; (2) thromboelastography (TEG) reports, including the responding time (R), *α* angle, and maximum amplitude (MA), showed abnormal coagulation system (*R* > 10 min, *α* angle < 50°, or MA < 50 mm represented insufficient coagulation factors, fibrinogen, and platelets, respectively) (3); (3) the PLT < 100 × 10^9^/L; (4) hemoglobin < 80 g/L; (5) with severe organ dysfunction; (6) with acute myocardial infarction; (7) refusal to consent and (8) with severe unstable hemodynamics before surgery. The sketch design of the study was shown in Figure 1.

### Anesthesia management

Once the patient was transferred to the operating room, a range of monitoring measures were applied including standard 5-lead electrocardiogram, pulse oxygen saturation, invasive blood pressure of the left radial and left femoral arteries, central venous pressure, bispectral index, regional cerebral oxygen saturation, nasopharyngeal and rectal temperature, etc. A binary catheter was inserted into right internal jugular vein for aPRP apheresis and fluid infusion. Usually, pre-CPB hematocrit (HCT) was maintained over 25%, between 20% and 23% during CPB and above 27% at the end of operation. The allogeneic blood transfusion was determined by anesthetist during operation and by the intensivist in intensive care unit (ICU) according to the criterion above. Generally, the requirement for the blood component transfusion was guided by TEG and laboratory reports except the above standard.

### aPRP separation procedure

The aPRP apheresis was performed with a blood component separation machine (Fresenius Kabi AG, Germany). Once the puncture of internal jugular vein was finished, blood was drawn from the central venous by the machine pump and processed with the centrifuge chamber into aPRP component and red blood cells (RBCs). RBCs were re-infused automatically using an autologous transfusion system and aPRP component was collected in a citrate bag. The harvested aPRP was stored at room temperature on a shaking tray to prevent the PLT from aggregation until the re-infusion. Usually, the aPRP apheresis procedure needed 58 ± 12 min and ceased at the time of the heparinization. According to our experience, about total 30–35 ml/kg with average whole blood volume 2,030 ± 250 ml was processed by the aPRP instrument. The harvested aPRP of each patient was approximate 703 ± 29 ml with the PLT concentration 264 ± 32 × 10^9^/L. During the collection of aPRP, blood volume was replaced by crystal solution, artificial colloid and 20% human albumin to maintain haemodynamics stability. Once aPRP apheresis was completed, CPB was started after heparinization. Perioperative laboratory data were collected, including PLT count, Mean Platelet Volume (MPV)/PLT ratio, TEG, C-reactive protein (CRP) and interleukin-6 (IL-6) levels before operation, during operation (after the transfusion of aPRP or allogeneic PLT), and 24 h and 8 days after operation.

### Aortic arch repair procedure

All surgical procedures in this study were performed by the same surgical team at our center. CPB was established by arterial perfusion of right axillary artery (RAA) and femoral artery (single pump double tubes perfusion style) and venous drainage of superior and inferior vena cava. Cardiac arrest was achieved by the retrograde perfusion of Custodiol HTK solution. The nasopharyngeal and rectal lowest temperature were around 27°C and 30°C, respectively. Circulatory arrest of lower body was conducted with bilateral cerebral perfusion through the RAA and the left common carotid artery. Meanwhile, the regional cerebral oxygen saturation was kept over 80% of baseline level. First, trunk stent-graft was inserted into the true lumen of the descending aorta. Then, careful anastomosis was performed between the proximal end of stent-graft and the distal end of four-branched graft. MHCA was ceased as soon as the anastomosis was finished and the duration was usually between 18 and 21 min. The left subclavian artery and left common carotid artery was reconstructed in sequence and body warming was started. Anastomosis was carried out between the main body of four-branched graft and the root of ascending aorta. Afterwards, the cross-clamp was removed and cardiac arrest was terminated. Then, innominate artery was reconstructed with 10 mm branch of aortic graft. At the end of CPB, nasopharyngeal and rectal temperature were around 36.5°C and 35.5°C, respectively. Hemostasis and sternal closure were conventionally conducted and patients were transferred to cardiac ICU postoperatively.

### Related definitions

Drainage volume was defined as the liquid flowed from the chest and pericardial drainage tube 24 h postoperatively. Perioperative blood transfusion meant the total amount of intraoperative and postoperative transfused allogeneic blood products. Postoperative neurological complications consisted of transient neurological deficit (TND) and permanent neurological deficit (PND) (24). All neurological complications were diagnosed with the consultation of an experienced neurologist who was blinded about this study. According to the urine output and serum creatinine levels, we determined whether continuous renal replacement therapy (CRRT) was needed. The hypoxemia was described on the basis of arterial oxygen partial pressure < 60 mmHg, prolonged ventilator support time (>3 days). The poor wound healing was recorded as undesirable healing of wound, sternum splitting, and substernal infection needed re-exploration. Preoperative laboratory tests were obtained as soon as the patients admitted to hospital, and intraoperative measurement meant the data after the transfusion of aPRP or allogeneic blood products before the returning to cardiac ICU. In addition, the total cost of blood products was obtained from blood bank, including the direct consumption of blood products and disposable blood conservation materials.

### Statistical analysis

All data were analyzed by using SPSS 20.0 (IBM, Armonk, NY). Continuous data were expressed as the mean ± standard deviation (M ± SD) or median with interquartile range, and Student *t* test was used for comparison of the independent sample. Categorical data were expressed as counts and percentages, and Pearson *χ*^2^-square was used for statistics. A *P* value < 0.05 was considered as statistically significant.

## Results

### Preoperative characteristics

The preoperative characteristics of patients were shown in Table 1. The demographics including age, gender and body surface area were similar in the two groups. There were no statistical differences in the preoperative TEG and the laboratory reports between the two groups. There were no significant differences in the two groups about the preoperative pulmonary related indexes including the arterial oxygen saturation, arterial oxygen pressure, and smoking history (Table 1).

**Table 1:** Demograhic and preoperative characteristics of the patients.

Variables	aPRP Group (*n* = 185)	Non-aPRP Group (*n* = 187)	*P* value
Gender (male)	126 (68.1%)	119 (63.6%)	0.424
Age	55.2 ± 7.4	55.9 ± 8.1	0.385
Body surface area (m^2^)	1.75 ± 0.32	1.74 ± 0.34	0.769
History of smoking	91 (49.2%)	89 (48.0%)	0.838
History of drinking	119 (64.3%)	108 (57.8%)	0.233
Arterial Sat O_2_ (%)	92.3 ± 5.3	93.1 ± 4.9	0.131
Arterial PaO_2_ (mmHg)	79.7 ± 9.3	79.3 ± 6.3	0.627
LVEF (%)	47.1 ± 3.9	47.9 ± 4.1	0.055
Mitral valve disease	11 (5.9%)	13 (7.0%)	0.854
Atrial fibrillation	8 (4.3%)	7 (3.7%)	0.983
Hematocrit	40.4 ± 7.9	39.8 ± 8.1	0.470
Diabetes mellitus	43 (23.2%)	49 (26.2%)	0.588
Time span from admission to operation (h)	5.7 ± 2.9	5.9 ± 3.3	0.535
D-dimer (μg/L)	4,360.4 ± 2,698.3	4,449.1 ± 2,577.5	0.746

LVEF, left ventricle ejection faction.

### Intraoperative data

The intraoperative data was shown in Table 2. One death in aPRP group and two in Non-aPRP group (*P* = 0.993) were observed during the operation. Another three patients (one in aPRP group and two in Non-aPRP group, *P* = 0.993) needed extracorporeal membrane oxygenation (ECMO) support to wean from CPB and survived. There were no significant differences in circulation arrest time, aortic cross clamp time and CPB time between the groups. Compared with Non-aPRP group, total operation time in aPRP group was shorter (*P* < 0.001). Intraoperative blood loss in aPRP group was significantly decreased compared with Non-aPRP group (*P* < 0.001). More human albumin was administrated in aPRP group than that in the Non-aPRP group (*P* < 0.001).

**Table 2:** The intraoperative profiles in the two groups.

Variables	aPRP Group (*n* = 184)^a^	Non-aPRP Group (*n* = 185)^a^	*P* value
ECMO Support	1 (0.5%)	2 (1.1%)	0.993
Cardiopulmonary bypass time (min)	218.4 ± 19.9	221.9 ± 28.1	0.167
Aortic cross clamp time (min)	136.5 ± 24.7	133.4 ± 21.9	0.201
Circulation arrest time (min)	19.9 ± 3.2	20.4 ± 1.9	0.068
Operation time (min)	301.1 ± 21.3	318.1 ± 29.9	<0.001
Human Albumin (ml)	309.4 ± 21.5	208.4 ± 50.9	<0.001
Cooling time (min)	51.2 ± 10.9	49.4 ± 13.9	0.166
Warming time (min)	66.9 ± 19.9	68.4 ± 15.6	0.419
Coronary artery bypass graft	26 (14.1%)	29 (15.6%)	0.803
Mitral valve replacement	11 (5.9%)	13 (7.0%)	0.854
Bentall + AAR + TSI	68 (37.0%)	70 (37.8%)	0.842
Aortic root reinforcement + AAR + TSI	108 (58.7%)	109 (58.9%)	
David + AAR + TSI	8 (4.3%)	6 (3.2%)	
Lowest nasopharyngeal temperature	26.8 ± 1.6	26.5 ± 1.9	0.100
Lowest rectal temperature	29.8 ± 2.9	29.4 ± 2.4	0.148
Blood loss (ml)	637.2 ± 24.9	908.4 ± 51.0	<0.001

ECMO, extracorporeal membrane oxygenation; AAR, aortic arch repair; TSI, trunk stent-graft implantation.

^a^ Exclude the intraoperative deaths.

### Postoperative outcomes

The postoperative outcomes of patients were shown in Table 3. During the postoperative period, 9 patients in aPRP group and 10 in Non-aPRP group died (*P* = 0.990), of which 16 deaths occurred within 8 days after operation and 3 more than 8 days. Causes of death included multiple organs failure, postoperative intractable cardiac dysfunction and refractory pulmonary complications. The extubation rate within 24 h in aPRP group was higher than that in Non-aPRP group (*P* < 0.001), and the incidence of postoperative hypoxemia [defined as in prior study (12)] was lower in aPRP group (*P* = 0.027). Mechanical ventilation time, the incidence of tracheotomy and the ICU duration in aPRP group were decreased (Table 3). Postoperative drainage volume in aPRP group was decreased compared with Non-aPRP group (*P* < 0.001). Furthermore, the incidence of re-operation due to excessive drainage in aPRP group was lower compared with the Non-aPRP group (*P* = 0.033). There were no significant differences in the rates of new-onset atrial fibrillation and other complications including TND, PND and renal dysfunction between the two groups. ICU stay was shorter in aPRP group, but the hospitalization duration was similar to Non-aPRP group. The hospitalization cost in aPRP group was much less compared with the Non-aPRP group (*P* < 0.001).

**Table 3:** Postoperative outcomes of the patients.

Variables	aPRP Group (*n* = 184)^a^	Non-aPRP Group (*n* = 185)^a^	*P* value
Mortality	9 (4.9%)	10 (5.4%)	0.990
Ventilator time (h)	25.3 (19.2, 37.0)	31.9 (25.4, 43.1)	<0.001
Extubated within 24 h	88 (47.8%)	31 (16.7%)	<0.001
Tracheotomy	4 (2.2%)	10 (5.4%)	0.176
Reintubation	3 (1.6%)	7 (3.8%)	0.341
Hypoxemia	15 (8.2%)	30 (16.2%)	0.027
Re-operation	2 (1.1%)	9 (4.9%)	0.033
Postoperative drainage volume (ml)	349.3 ± 111.5	589.7 ± 147.2	<0.001
Poor healing of wound	5 (2.7%)	7 (3.8%)	0.776
Acute renal dysfunction	29 (15.8%)	39 (21.1%)	0.237
Need for dialysis	19 (10.3%)	27 (14.6%)	0.279
TND	36 (19.6%)	39 (21.1%)	0.816
PND	6 (3.3%)	7 (3.8%)	0.992
New-onset AF	4 (2.2%)	5 (2.7%)	0.993
Intensive care unit stay (day)	4.8 (3.5, 7.9)	8.7 (4.9,11.2)	<0.001
Stay of hospital (day)	15.0 (11.4, 20.0)	17.1 (14.2,22.1)	0.089
Discharged voluntary	7 (3.8%)	7 (3.8%)	0.793
Hospitalization costs (thousand yuan, RMB)	243.5 ± 33.1	297.6 ± 43.5	<0.001

PND, permanent neurological deficit; TND, transient neurological deficit; AF, atrial fibrillation.

^a^ Exclude the intraoperative deaths.

### Perioperative laboratory measurements

**Figure 2:**
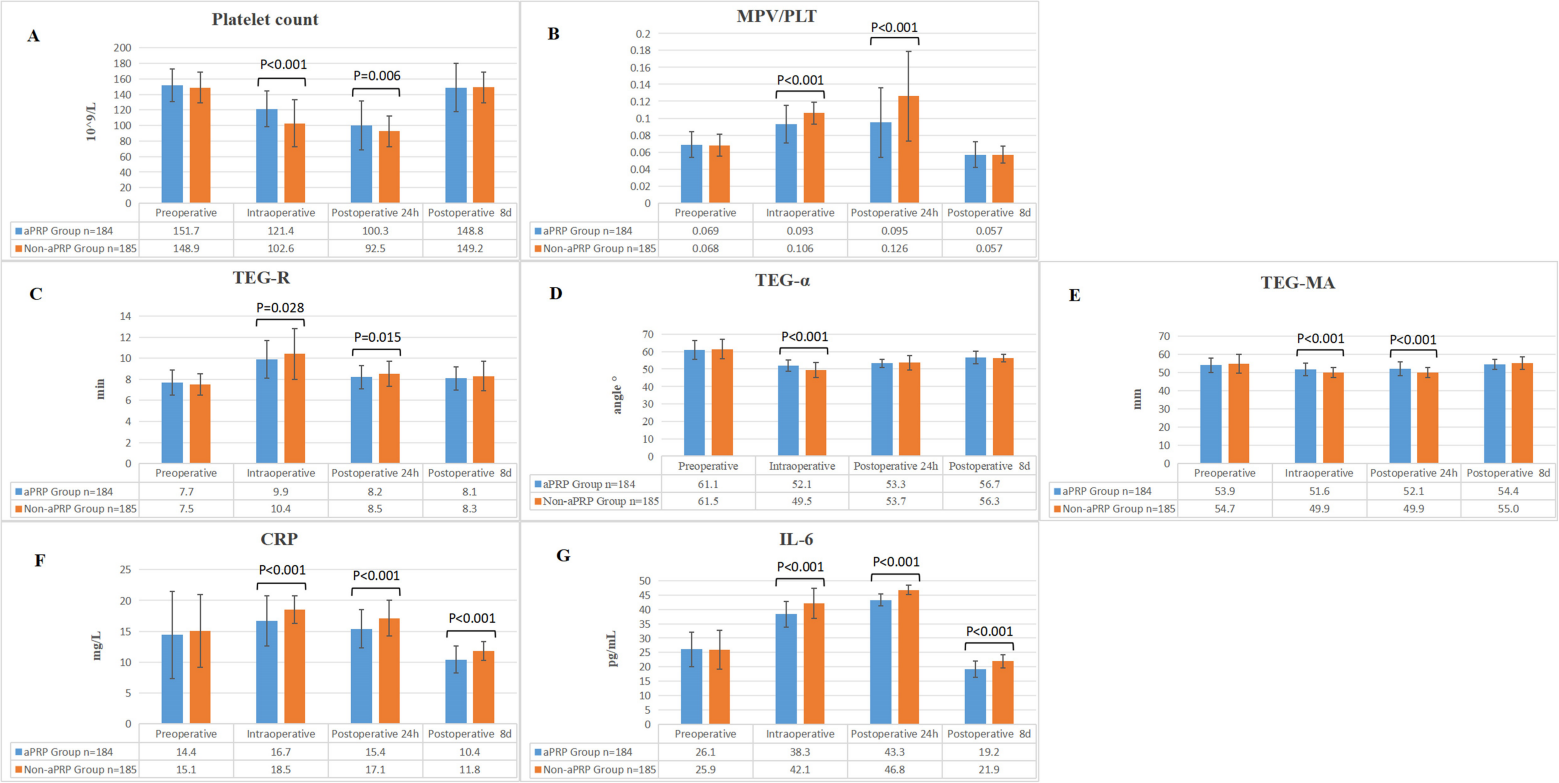
Perioperative laboratory examinations of the two groups. **(A)** Comparison of PLT count between the two groups. **(B)** The ratio of MPV/PLT in the two groups. **(C)** The trend of TEG-R in the two groups. **(D)** The change tendency of TEG-α in the two groups. **(E)** The dynamic changes of TEG-MA in the two groups. **(F)** The numerical variations in CRP between the two groups. **(G)** Comparison of IL-6 levels between the two groups. aPRP, autologous platelet rich plasma; PLT, platelet; MPV, Mean Platelet Volume; TEG, thromboelastography; CRP, C-reactive protein; IL-6, interleukin-6.

No hemodynamic instability was noted during aPRP harvesting. Perioperative laboratory measurements were compared (Figure 2) and there were no significant differences in all the preoperative measurements between the two groups, including PLT, MPV/PLT, TEG, CRP and IL-6. The intraoperative PLT count, TEG-α and TEG-MA in aPRP group were higher than that in Non-aPRP group, while MPV/PLT and TEG-R were lower. At 24 h after operation, PLT count and TEG-MA value in aPRP group were still higher, and MPV/PLT and TEG-R were lower than that in Non-aPRP group. The CRP and IL-6 levels of aPRP group were lower compared with Non-aPRP group during intraoperative period, as well as at 24 h and 8 days postoperatively (Figure 2).

### Consumption of application of blood products

Perioperative application of allogeneic blood products was shown in Table 4. The transfusion of allogeneic PLT, plasma, RBC and cryoprecipitate were decreased in aPRP group compared with the Non-aPRP group, respectively. The total cost of blood consumption was much lower in aPRP group than that in the Non-aPRP group (*P* = 0.003).

**Table 4:** Application of allogeneic blood products and blood costs.

Blood component	aPRP Group (*n* = 184)^a^	Non-aPRP Group (*n* = 185)^a^	*P* value
Platelet (therapeutic dose)	2.11 ± 1.03	2.52 ± 0.83	<0.001
Plasma (ml)	405.6 ± 55.6	421.0 ± 61.7	0.012
Cryoprecipitate (U)	9.7 ± 2.4	10.4 ± 1.9	0.002
Red blood cells (ml)	422.7 ± 64.9	479.2 ± 81.0	<0.001
Blood consumption (yuan, RMB)	9,202.2 ± 1,597.4	10,031.9 ± 3,471.8	0.003

^a^ Exclude the intraoperative deaths.

## Discussion

The present study demonstrated that patients undergoing Sun's procedure with MHCA could obtain benefits from the preoperative aPRP collection and re-infusion. First, this technique reduced the intraoperative blood loss and allogeneic blood transfusion. Secondly, aPRP collection and re-infusion decreased the postoperative drainage and pulmonary related complications, and subsequently reduced the ICU duration and hospitalization cost. Finally, this study indicated that aPRP collection and re-infusion alleviated postoperative inflammation and improved the early outcome of Sun's procedure. However, results also suggested that aPRP technique did not influence the perioperative mortality, the hospital stay, the incidences of postoperative renal dysfunction and cerebral complications.

It is acknowledged that the reduced PLT count and impaired function are the leading cause and independent risk factors for bleeding in cardiovascular surgery with CPB. Previous reports have indicated that aPRP collection could minimize the post-CPB bleeding and subsequently reduced the blood transfusion during cardiac surgery (3, 18). It did this by providing patients with autologous integrated PLT. However, a handful of studies insisted on the opposite view (25, 26). The controversy may arise from the fact that enough aPRP helps to reduce bleeding and insufficient dose of PLT may actually have negative effects (27). By general consent, extracted aPRP with over 20% of blood volume is conducible to the coagulation functional improvement (28). In our study, the average volume of blood collection was more than 20% of average blood volume and the harvested PLT concentration (264 ± 32 × 10^9^/L) was nearly close to the adequate number (260–300 × 10^9^/L) according to the literature (28). Apart from the yield of PLT, transfusion protocols may also be the reason that aPRP in earlier studies demonstrated negative results. In our study, the transfusion protocols had the same standard and the study was conducted in a single center, which could diminish the variations in some degree.

During the procedure of aPRP apheresis, crystal and colloids fluids were infused for the complement of blood volume according to the blood pressure and central venous pressure to maintain the hemodynamic stability. Human albumin was a good choice to balance the colloid osmotic pressure because plasma was temporarily lost in the process of aPRP separation. Therefore, perioperative usage of human albumin in aPRP group was much more than that in Non-aPRP group. If emergency CPB was required due to changes in the condition, for example, aortic dissection rupture, the aPRP collection must be terminated. However, no case of hemodynamic instability was found during aPRP collection in the current study. Storage of the harvested aPRP at room temperature on a shaking tray is optimal for preserving PLT function. The shorter period that the PLT is stored, the lower risk that bacterial contamination or replication is involved (28). However, whether the apheresis process itself activates PLT like during CPB and whether the storage of PLT in citrate bag damages the PLT membrane were still in debate. Even though the morphological structure of aPRP was not investigated in our study, we thought the aPRP function could not be judged from this aspect merely. Both the laboratory reports of PLT indices and the clinical results demonstrated a satisfactory outcome. Another simultaneous phenomenon during aPRP apheresis and re-infusion was hypocalcemia due to the application of citrate. This problem could be resolved easily by the supplement of calcium preparations.

The process of aPRP collection is usually considered time-consuming and prolongs the operation time. In our study, the aPRP extracting started immediately after the internal jugular vein puncture. At the meanwhile, the surgeons separated the peripheral arteries (femoral and axillary artery) and three branches of the arch. The aPRP apheresis was nearly completed before the heparinization without wasting any additional operation time. In contrast, the whole operation time in aPRP group was shorter than in Non-aPRP group due to the shorter hemostasis time.

We chose MPV/PLT as an indicator of PLT activation because of its increased clinical value than independent MPV or PLT (29). Once PLT is activated, its shape changes from disc-shaped to irregular, and pseudopods form on their surface, leading to an increase in MPV (30). The aggregation of PLT depends on the bridging of these pseudopods. Therefore, elevated MPV level is associated with enhanced PLT aggregation ability (31). The extensive consumption of PLTs due to their aggregation results in a decreased peripheral blood PLT count. During operation (after transfusion of aPRP) and at the postoperative 24 h, the higher PLT count and lower MPV/PLT in aPRP group indicated the less activation of PLT than the Non-aPRP group. In other words, aPRP supplied the patient with more functional PLT which avoided the destroy of CPB. As a result, the bleeding or errhysis in the aPRP group was treated more easily than the Non-aPRP group. It is said that the life cycle of PLT is 8–10 days and about 10% new PLT supplement enters the circulation every day (32). This could account for the results that PLT count and MPV/PLT have been recovered to the preoperative levels until the 8th postoperative day.

As we know, the adhesion of PLT to the non-physical lumen not only activates the PLT itself but also triggers the release of coagulation factors from the activated PLT, including fibrinogen, Von Willebrand factor and factor V. TEG is a novel method for detecting coagulation function, serving as an important reference for guiding blood transfusions and assessing coagulation status (33). TEG-R measures the time required to form the first detectable blood clot. TEG-MA reflects the maximum hardness of the blood clot and PLT function. TEG-α primarily indicates fibrinogen function. The TEG results demonstrated that aPRP apheresis preserved the function of PLT, coagulation factors and fibrinogen, subsequently improved the coagulation and hemostasis. We could deduce that conserving the integrity of PLT might also preserve the coagulation factors, and the integrated autologous PLT might be more effective than the allogeneic PLT in improving coagulation function.

As a critical trigger of inflammation, the activation of PLT plays an important role in the initiation and progression of inflammation due to the release of pro-inflammatory cytokines (34). There is evidence that activated PLT plays an important role in acute and chronic inflammation by interacting extensively with leukocytes and endothelial cells (35). CRP, a nonspecific inflammatory marker, is an important predictor and prognostic indicator in AAD patients (36, 37). Existing studies suggest that there was a positive correlation between CRP and PLT activation in AAD (29). The synthesis and release of CRP is regulated by IL-6 (38, 39). Although CRP is released in response to significant pro-inflammatory stimuli, this process is closely related to circulating IL-6 levels (40). Our results showed the decreased CRP and IL-6 levels in the aPRP group during intraoperative and postoperative period. We speculated that the apheresis of aPRP before CPB might alleviate the inflammatory response by decreasing the activation of PLT to some extents.

Similar to other reports (3, 18), aPRP reduced the incidences of pulmonary related complications such as tracheotomy, reintubation and hypoxemia in our study. The low incidences of pulmonary complications contributed to the shortened mechanical ventilation time and ICU stay in aPRP group. It was worth emphasizing that under the unified standard management of the ICU director, the two groups of patients adopted the same extubation protocol and ICU management. The extubation rate within postoperative 24 h in aPRP group in our study was lower than that in Zhou's study (19) (47.8% vs. 81.6%). We thought such difference might be caused by the different physical characteristics of the patients, the sample size and the extubation routine of the intensivist. In addition, occurrence of re-operation for excessive drainage in the Non-aPRP group was higher compared with the aPRP group. This results also confirmed the fact that aPRP technique in Sun's procedure reduced the intraoperative bleeding and the postoperative drainage. However, no evidence showed that aPRP could decrease postoperative mortality and other complications.

It has been proven that huge infusion of allogeneic blood products could increase respiratory complications (12, 41). This was especially true for patients who already suffered pulmonary inflammation at the onset of AAD. The results in the current study demonstrated that intraoperative bleeding or errhysis (637.2 ± 24.9 vs. 908.4 ± 51.0 ml), postoperative drainage (349.3 ± 111.5 vs. 589.7 ± 147.2 ml), and perioperative blood transfusion (Table 4) in aPRP group were decreased. We also found that pulmonary complications, ICU duration and hospitalization cost (Table 3) were significantly reduced in aPRP group. We could deduce that the less bleeding and transfusion facilitated the low incidence of pulmonary complications and the shorter ICU stay. In other words, the huge infusion of the allogeneic blood products might explain the high incidence of pulmonary complications and the high inflammation index in the Non-aPRP. Hospitalization cost of AAD patients in China is mainly spent on operation and the treatment during ICU stay. Although aPRP apheresis increased some cost, the average hospitalization cost in the aPRP group was lowered by about 60 thousand yuan (RMB) compared with the Non-aPRP group. This reduction was attributed to the less blood loss, reduced transfusion, decreased pulmonary complications and ICU stay. Unlike other reports (18, 19), the hospital stay was not shortened by the aPRP application in our study. It was because some patients in the aPRP group who met the discharge criteria believed that extending hospital stay would be more beneficial for their recovery, and we tried our best to respect their own wishes. We thought it was still a controversial issue in different countries.

There were several limitations in our study. Firstly, the integrity and function of the harvested aPRP were not proven by the morphological and microscopic features, that might be a more convincing evidence for the efficacy of aPRP. Secondly, during the aPRP collection process, we used fluid supplementation to maintain hemodynamic stability in patients, but the inconsistency in the types of fluids or blood products administered to different patients introduces a confounding variable. Thirdly, emergency operation involved many uncontrollable factors such as the economic reason, wills of the patients' relatives and religions, and the control of liquid infusion in the two groups, which might influence the results. Furthermore, the CRP and IL-6 level might be affected by multiple transfusions. This study is retrospective and is therefore subject to the inherent limitations. Although the intensive care physicians were blinded to the patients, but it was difficult for the surgeons because the aPRP was carried out in the operating room. Finally, this study was completed in a single center and the long-term outcome was not reported. We will continue to follow up with the patients in the further study.

## Conclusion

To sum up, application of aPRP technique reduced the blood loss and allogeneic blood transfusions in Sun's procedure under MHCA. The aPRP apheresis and re-infusion decreased the postoperative pulmonary complications, shortened the ICU stay and reduced the hospitalization cost, but did not have impact on the occurrence of postoperative renal function, cerebral complication and hospitalization stay.

## Data Availability

The original contributions presented in the study are included in the article/Supplementary Material, further inquiries can be directed to the corresponding author.
